# Vancomycin Loaded Glycerol Monooleate Liquid Crystalline Phases Modified with Surfactants

**DOI:** 10.3390/pharmaceutics12060521

**Published:** 2020-06-08

**Authors:** Spomenka Milak, Angela Chemelli, Otto Glatter, Andreas Zimmer

**Affiliations:** 1 Department of Pharmaceutical Technology and Biopharmacy, Institute of Pharmaceutical Sciences, University of Graz, NAWI Graz, Universitätsplatz 1, 8010 Graz, Austria; spomenka.milak@uni-graz.at; 2 Institute of Inorganic Chemistry, Graz University of Technology, 8010 Graz, Austria; angela.chemelli@tugraz.at (A.C.); otto.glatter@uni-graz.at (O.G.)

**Keywords:** lyotropic mesophase, vancomycin hydrochloride, encapsulation, glycerol monooleate, tuning agent, polyglycerol ester, triblock copolymer, eye infections

## Abstract

The influence of two tuning agents, polyglycerol ester (PE) and triblock copolymer (TC), on the properties of glycerol monooleate (MO) liquid crystalline phase (LCP) was investigated to achieve the therapeutic concentration of vancomycin hydrochloride (VHCl) into the eye, topically during 60 min (1 h) and intravitreally during 2880 min (48 h). Different techniques were used to elucidate the impact of surfactants on the structure of the LCP: polarized light microscopy (PLM), small-angle X-ray scattering (SAXS), and in vitro release tests I and II (simulating local and intravitreal application in the eye). The structure analysis by SAXS depicts that the inclusion of PE into the MO LCP provided partial transition of a hexagonal phase into a lamellar phase, and TC induced a partial transition of a hexagonal phase into an LCP which identification was difficult. The LCP modulated with PE and TC demonstrated different VHCl’s release patterns and were evaluated by comparing our release data with the literature data. The comparison indicated that the LCP modulated with 30% *w*/*w* PE could be a promising VHCl delivery system intravitreally during 2880 min.

## 1. Introduction

Over the past decades, the lyotropic liquid crystalline phases (LCPs) made of polar lipids have received increased attention for controlled drug release [1]. The highly ordered internal structure, possibility of co-existing at thermodynamic equilibrium in excess water conditions, and the possibility to encapsulate different molecules led to interesting release properties of such delivery systems [1,2,3,4,5]. Glycerol monooleate (MO) is one of the most investigated amphiphilic lipids forming LCPs for the controlled release of target drugs, primarily due to its polymorphism and ease of accessibility. When placed in a water solution, MO gives rise to several phases with one-, two-, or three-dimensional periodicities, ranging from reverse micelle to a lamellar phase, reversed bicontinuous cubic LCPs (cubic phase) of different symmetries (*Ia3d* and *Pn3m*), and reversed hexagonal LCPs (hexagonal phase) [6]. The cubic phase is a three-dimensional phase composed of a continuous fluid lipid bilayer separating two interwoven continuous water networks (the diameter of the fully swollen aqueous channel is about 50 Å [7,8]). The hexagonal phase is composed of densely packed, infinitely long and straight water-filled rods [6]. The hexagonal phase has lower viscosity compared to the relatively stiff cubic phase making its practical application easier.

The unique nanostructure of LCPs partially controls the drug release kinetics, where the drug has to diffuse through the internal nanostructure to access the external environment [2,3,4]. Furthermore, the location of the drug, its size, and polarity are important parameters affecting the diffusion and release rate. For hydrophobic drugs that are embedded in the lipid bilayer of the LCP, release kinetics depend on the partitioning between the lipidic and aqueous compartments. In contrast, hydrophilic drugs reside preferentially in the aqueous channels. The release of hydrophilic drugs dissolved within the aqueous domains of the different LCPs depends very much on the liquid crystalline internal structure. In the case of hydrophilic drugs, the release from cubic phases can take place without having to cross the lipid bilayer, and thus release occurs at a much faster rate [2,9]. The release of hydrophilic drugs from the hexagonal phase takes place through discontinuous (closed) water rod-like compartments. This occurs by crossing the lipid bilayer following the slower release pattern than by the continuous water channels of the cubic phase. Among LCPs, much attention has been focusing on the cubic phase and its release pattern investigations [1,3,4].

The complex structure of LCPs is consequently dependent on various factors ranging from the molecular shape and concentration of lipids to environmental conditions such as temperature, pressure, pH [10,11]. Several studies have been run to investigate the possibility to control the drug diffusion rate by intrinsically manipulating the structural symmetry of the LCP. There are various approaches being developed for controlling drug transport properties and engineering drug diffusion rates in LCPs in response to either endogenous or exogenous stimuli [12]. Endogenous stimuli could be the pH [13,14,15,16,17], using ionic interactions for the controlled entrapment and drug release [18,19,20,21], controlled diffusion by modulation of hydrophobic interactions [22] and controlled diffusion by modulation of LCP channel size [23,24,25]. The inherent properties of LCPs could be altered further by exogenous stimuli such as temperature [26,27,28], pressure, light [26,29,30,31,32,33,34], magnetic or electric field [35,36].

Recently, our group has investigated the vancomycin hydrochloride (VHCl) release properties from the MO swollen cubic and hexagonal phases to reach the desired ocular VHCl concentration topically or intravitreally [37]. The hexagonal phase demonstrated very slow (2.73 ± 0.3%) VHCl release during 1440 min simulating the local ocular administration. The insert prepared from the cubic phase did not reach the desired ocular VHCl concentration topically (in vitro VHCl release test I) and should be further optimized to be effective topically into the eye. However, the cubic phase demonstrated that the effective dose was delivered into a simulated intravitreal tissue [37]. As the hexagonal phase revealed the VHCl’s sustained release, further investigations have been done on the hexagonal phase and modulation of its structure in an attempt to achieve the desired therapeutic concentration of VHCl topically or intravitreally into the eye. It is assumed that VHCl in the hexagonal phase was located mostly in one-dimensional parallel water cylinders. Therefore, the pathway to enhance the therapeutic efficacy of VHCl in the hexagonal phase could be through controlling VHCl transport properties and affecting its diffusion rate in response to an additional swelling agent in the water domain. The control over the VHCl diffusion rate will be done by intrinsically manipulating the structural symmetry of the MO LCP using polyglycerol ester (PE) and triblock copolymer (TC) (hydration enhancing agents) as tuning agents. It is to expect that large hydrophilic molecules such as TC will compete with the hydration water of the hydrophilic glycerol group of the MO, cause bending of the interfacial film toward the water, and accordingly, shrink the LCP. The derivation of fatty acids with larger hydrophilic head groups such as PE would have an opposite effect on the interfacial film curvature promoting increased water capacity and increased structural parameters. The goal of this study was to develop a VHCl-loaded liquid crystalline insert from a bulk LCP modulated with tuning agents, and examine its ability to maintain the therapeutic VHCl concentration during 60 min in the eye topically and intravitreally during 2880 min in the management of eye infections.

## 2. Materials and Methods

### 2.1. Materials

MO (MONOMULS 90-O 18 PH) was a gift from Cognis GmbH, Monheim am Rhein, Germany and used as received. VHCl was provided by Alpharma Taizhou Pharmaceuticals Co. Ltd., Zhejiang, China. Paraffin oil (PO) was purchased from Herba Chemosan ACM, Graz, Austria. The structure of the bulk LCP was modulated, adding the following surfactants: PE or TC. PE (trade name GRINSTED^®^ PGE 308/D KOSHER from Danisco A/S, Braband, Denmark) is a mixture of polyglycerol esters made from fatty acids, in which the polyglycerol moiety is mainly mono-, di-, tri-, and tetraglycerols. PE was a gift from a Danisco A/S, Braband, Denmark. TC (trade name Lutrol^®^ F 127 from BASF SE, Limburgerhof, Germany) is a triblock copolymer of ethylene oxide and propylene oxide and was a gift from BASF SE, Limburgerhof, Germany. Trimethylamine (≥ 99.5%) were purchased from Sigma-Aldrich Chemie, Steinheim, Germany. Acetonitrile, dichloromethane and tetrahydrofuranol of HPLC grade were purchased from VWR BDH Prolabo, Leuven, Belgium. The water used for the HPLC analyses and preparation of the release media was ultrapure water (Milli-Q^®^ Gradient water purification system, Millipore S.A.S., Molsheim, France). Water for injection, supplied by Fresenius Kabi Graz, Austria was used for preparation of the LCPs.

### 2.2. Methods

#### 2.2.1. Preparation of the Bulk LCPs

A melt homogenization method, as described in our previous work, [37] was applied in this study to further investigate the structure and release properties of the modulated LCP. The samples of bulk LCP modulated with PE were prepared in the following way: MO (33–53% *w*/*w*), PE (10–30% *w*/*w*) and PO (7% *w*/*w*) (keeping the constant ratio = (MO+PE):PO = 9:1) were melted at 40 ºC. The VHCl solution in water (9.5% *w*/*w*) was manufactured and filtered using a 0.2 µm membrane filter. The filtered VHCl solution was compounded with the MO–PE–PO melted mixture using the Cito Unguator^®^E 100 homogenizer (GAKO^®^ International GmbH, München, Germany).

The samples of bulk LCP modulated with TC were prepared in the following way: MO (63% *w*/*w*) and PO (7% *w*/*w*) (keeping the constant ratio = MO:PO = 9:1) were melted at 40 °C. TC (1–10% *w*/*w*) and VHCl (9.5% *w*/*w*) were dissolved in water for injection, filtered on a 0.2 µm membrane filter, and this solution was used in the above formulation as 30% *w*/*w*. The subsequent preparation procedure was the same as for the LCP modulated with PE. The samples of LCPs were prepared in triplicate.

The LCPs samples were prepared in aseptic conditions (Safety cabinet Typ KS12, Thermo Scientific Thermo Electron LED, Langenselbold, Germany). The autoclave (AutoklavTyp 26, Melag Medizintechnik, Berlin, Germany) was used to sterilize the laboratory glassware. The VHCl water solution, as mentioned previously, was filtered by the 0.2 µm membrane filter (Sterile Syringe Filter, 0.2 µm Cellulose Acetate, VWR International, Wien, Austria).

#### 2.2.2. Characterization of LCPs

##### Polarized Light Microscopy (PLM)

The first screening of the structure of bulk LCPs was done by PLM, discriminating isotropic lipid LCPs, such as the cubic, L_2_ (inverse micellar phase or so-called “emulsified microemulsion”), and L_3_ (bicontinuous fluid sponge phase) from anisotropic phases: the lamellar (L_α_) and the hexagonal (H_II_) [9,38].

The PLM analysis has been performed by a Zeiss microscope (“Axiovert” 40 CFL, HBO 50/AC, Carl Zeiss Meditec Co., Ltd., Japan) using cross-polarizers (Nikon Optiphot, Nikon, Japan) and connected to a digital Nikon D300 camera (Nikon, Japan) and a PC monitor.

##### Small Angle X-ray Scattering (SAXS)

SAXS analyses have been used to further investigate the structure of the LCP such as their identification and structure parameter comparison.

The source of X-ray is a sealed tube X-ray generator (DebyeFlex3000) that emits Cu-Kά radiation (λ = 0.154 nm). The SAXSess camera (Anton-Paar, Graz, Austria) is connected to an X-ray generator. The monochromatized X-rays are focused in vertical direction (Multilayer-mirror optics) and then confined by a vertical asymmetric Kratky slit collimation-system.

The scattering patterns were recorded by a Mythen 1K (Dectris, Switzerland) Detector (1280 pixels and 50 µm, active length: 6.4 cm). The scattering curves were recorded 10 times for 10 s each.

An angular range (2Q) of 0.1° to ~7° with respect to the incident beam obtained in the transmission mode was used to get information about the structures in the range from 1 to 100 nm.

A special paste holder has been used for the samples of bulk LCPs. The sample of bulk LCPs is sandwiched between two foils and is sealed vacuum tight. For the temperature control of the samples, a Peltier element was used. The samples were kept at 25 °C.

The SAXSquant software (Anton–Paar, Graz, Austria) was used for the control of the camera, measurements, and for generating scattering curves (1D) from the detector data.

The calculation of the radius of water channels (*r*) in the hexagonal LCP could be estimated as follows [37]:

(1) r=a(1−ϕ)12(32)12

where ϕ is the lipid volume fraction and a is the structure parameter as measured by SAXS [37].

##### In Vitro VHCl Release Test I

The in vitro VHCl release test I has been used to investigate the release properties of inserts (prepared by means of a syringe of 2 mm in diameter and 4 mm in length) in the freshly prepared simulated tear fluid (NaHCO_3_ 0.218 g/100.0 mL, NaCl 0.678 g/100.0 mL, CaCl_2_ × 2H_2_O 0.0084 g/100.0 mL, KCl 0.138 g/100.0 mL, Milli-Q^®^ water up to 100.0 mL; pH = 7.4) [39], simulating the administration in the eye topically. The trials have been done in triplicate, at 32 ± 0.5 °C, using 1.5 mL polypropylene tubes (Greiner Bio One GmbH, Frickenhausen, Germany) filled with 1 mL of the release medium and an insert (10–20 mg), shaken at 300 rpm by the Eppendorf Comfort thermomixer (Eppendorf AG, Hamburg, Germany).

The release pattern was investigated at the time point: 0, 5, 10, 30, 60, 120, 180, 360, 600, 1440, 2880 min, centrifuged (Centrifuge Eppendorf 5804R, at 24 °C, 14,000 rpm, 10 min) and analyzed using the later described HPLC method.

The Higuchi diffusion equation was used to check if the release pattern could be described by diffusion:

*Q* = *A*[*D*(2*C*−*C*_*s*_)*C_s_t*]^1/2^ (2)

where *Q* is the amount of the drug released in time *t* per unit area *A*, *D* is the diffusion coefficient of the drug molecules, *C* is the initial concentration of the drug, and *C_s_* is the solubility of the drug in the matrix media [40].

##### In Vitro VHCl Release Test II

**Figure 1:**
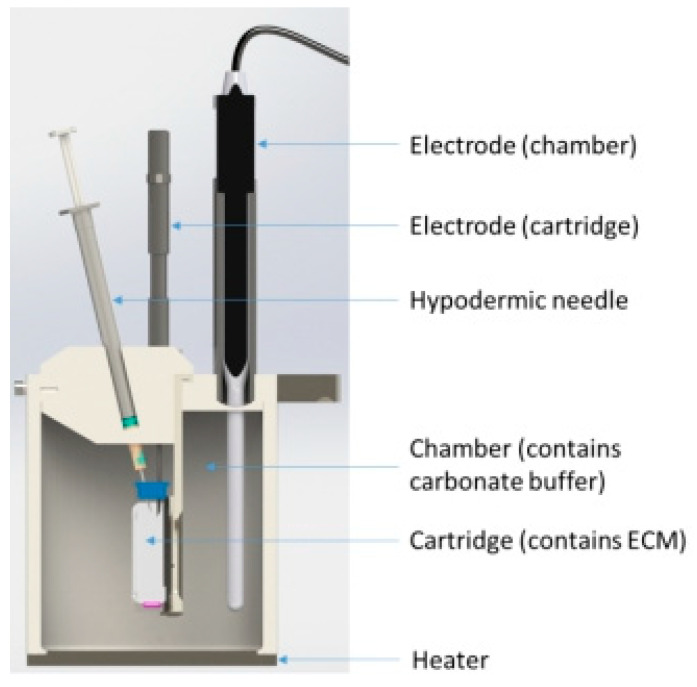
Scheme of the Sirius’ Subcutaneous Injection Site Simulator used for the in vitro VHCl release test II.

The in vitro release test II was done to simulate intravitreal ocular administration by a Scissor (Sirius’ Subcutaneous Injection Site Simulator manufactured by Sirius Analytical Instruments Ltd./Pion Inc., Forest Row, UK) (Figure 1). The scissor enables to investigate changes of a drug in the human body on the way to the target place such as the changes of pH, temperature, pressure, buffer, and tissue composition. Scissor consists of a cartridge (simulating the injection site) located in a chamber (simulating the interstitial fluid). The cartridge is filled with an artificial extracellular matrix (ECM) consisting of a hyaluronic acid gel (5 mg/mL). The chamber is filled with the 300 mL of carbonate buffer (pH = 7.4) (300 mL is an optimal volume of buffer recommended by the manufacturer). The cartridge has a membrane cutoff of 1,00,000 Da and allows diffusion of the formulation components (VHCl and excipients) into the chamber. The quantity of the sample injected is about 1 g. The concentration of VHCl solution was 6 g/100 mL, and the bulk LCP with PE contained 30% *w*/*w* of the VHCl solution 9.5% *w*/*w*. The stirring speed used was 200 rpm.

The permeation patterns of the two formulations of VHCl—the VHCl solution and the bulk LCP tuned with PE 30% *w*/*w* were investigated in this trial.

The concentration of the VHCl permeated into the carbonate buffer was measured by a reverse phase HPLC at the time points: 0, 60, 120, 180, 240, 360, 480, 960, 1440, 2160, 2880, 3600, and 4320 min.

##### High-Performance Liquid Chromatography (HPLC) Analysis of VHCl

The quality and quantity of VHCl were analyzed using a gradient reversed-phase HPLC according to the European Pharmacopoeia. This method was used to evaluate the amount of VHCl released from the LCPs tuned with PE or TC during the in vitro VHCl release test I and in vitro VHCl release test II.

The HPLC equipment used was from Agilent^®^ Technologies, Waldbronn, Germany. The HPLC equipment is assembled from an HPLC system (1260 Infinity Agilent^®^ Technologies, Waldbronn, Germany), a quaternary pump (1260 Bio Quat Pump, DEAB600659), a degasser, an ALS autosampler equipped with a G1330B 1290 thermostated column oven, and a diode-array detector set at 280 nm. An octadecylsilyl silica gel C18(2), 250 × 4.60 mm (5 µm) column has been used as the separation system. The 92% triethylamine buffer (with 6% phosphoric acid, pH = 3.2), 1% tetrahydrofuran, and 7% acetonitrile was used as the mobile phase A (equilibration buffer). The 70% triethylamine buffer (with 6% phosphoric acid, pH = 3.2), 1% tetrahydrofuran, and 29% acetonitrile were used as the mobile phase B (elution buffer). The gradient mode used 0% elution buffer at the start, and, after 26 min, ending with 100% elution buffer. The flow rate used was 1.0 mL/min and the temperature was 25 °C. A peak integration has been done by the OpenLAB CDSRev (C.01.05 Software Version, Agilent^®^ Technologies, Waldbronn, Germany, 2015). The VHCl concentration has been determined using a standard calibration curve at the investigated concentration range.

The linear standard calibration curves have been generated in the VHCl investigated concentration range of 0.01–0.25 mg/mL (the correlation coefficients were > 0.9994). The lowest VHCl concentration determined corresponded to 0.008 mg/mL.

## 3. Results

### 3.1. PLM

**Figure 2:**
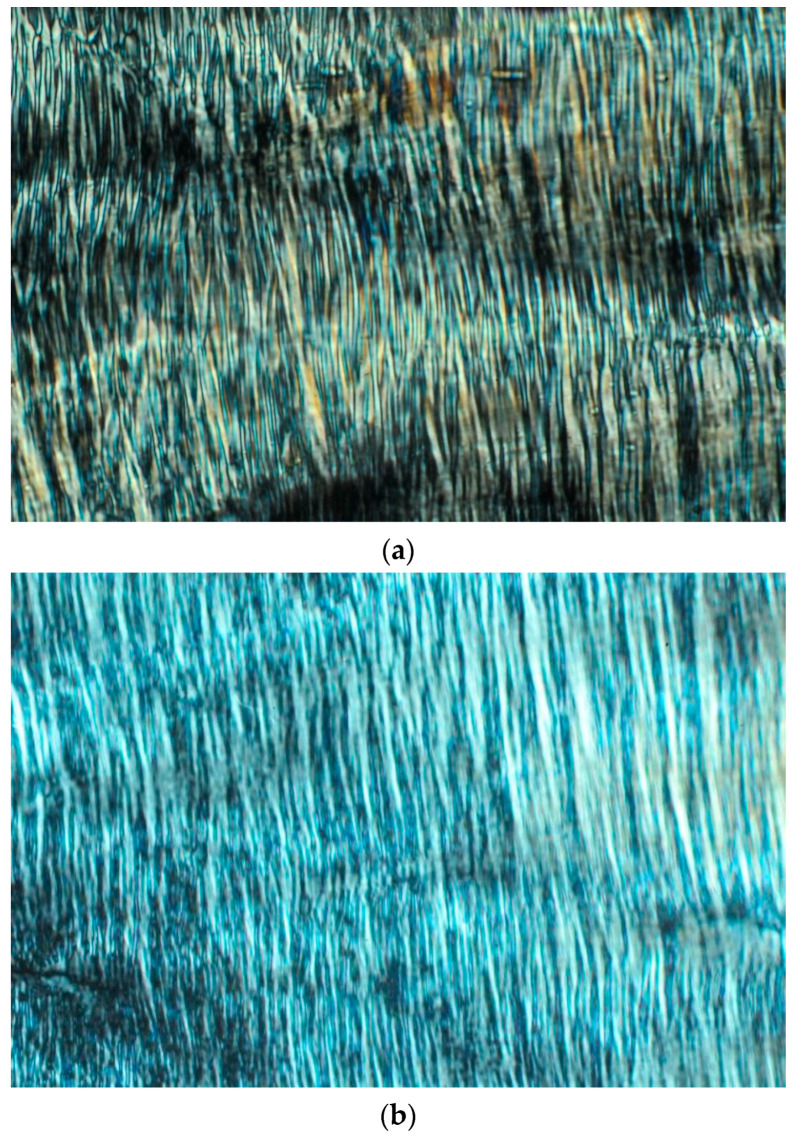
Polarizing light microscopy (**a**) MO 43% *w*/*w*, PE 20% *w*/*w*, PO 7% *w*/*w* and VHCl sol. 30% *w*/*w*; (**b**) MO 63% *w*/*w*, PO 7% *w*/*w* and (VHCl sol. 9.5% *w*/*w* and TC 10% *w*/*w*) 30% *w*/*w*. The magnification was 200×.

PLM images of the LCPs modulated with PE or TC are revealed in Figure 2. Both images displayed birefringent and colorful textures, typical fan-like structures, that could be attributed to the hexagonal symmetry [9,38].

### 3.2. SAXS

Table 1 presents the SAXS results of LCPs prepared with different conc. of PE or TC. The SAXS spectra of both LCPs, modulated with PE and TC, revealed the mixtures of the hexagonal phase with other LCPs.

SAXS results from the samples with PE point to the existence of two structures. The addition of 20–30% *w*/*w* PE enabled the determination of the formation of a lamellar phase. The spacing of the bilayers, as well as the structure parameter of the hexagonal phase, increased with increasing PE content. In the case of the lamellar phase, the addition of 10% *w*/*w* PE did not follow this trend. The SAXS patterns revealed the appearance of one additional peak, also indicating the formation of a second structure. This peak could correspond to a cubic *Ia3d* phase, which occurs if the water content is lower compared to the hexagonal phase [41]. 

The effect of increasing PE concentration and TC concentration on the radius of water channels calculated by using Equation (1) and the structure parameters determining using SAXS is summarized in Table 1. Increasing the PE concentration from 10–30% *w*/*w* induced a steady increase in the radius of water channels of the hexagonal phase, from 4.63 nm to 6.63 nm, following the behavior of the structure parameter.

The samples with TC revealed the structures of the hexagonal phase and an additional phase. Due to the unfortunate overlap of peaks as well as the lower portion of the additional phase at low TC content, the determination of the space group was difficult. Contrary to the addition of PE, TC decreased the hexagonal structure. The trend of decreasing the radius of water channels (from 3.81–3.43 nm) of the hexagonal phase in the samples with increasing TC concentration from 1–10% *w*/*w* following the behavior of the structure parameter (6.732–6.061 nm).

### 3.3. In Vitro VHCl Release Test I

**Figure 3:**
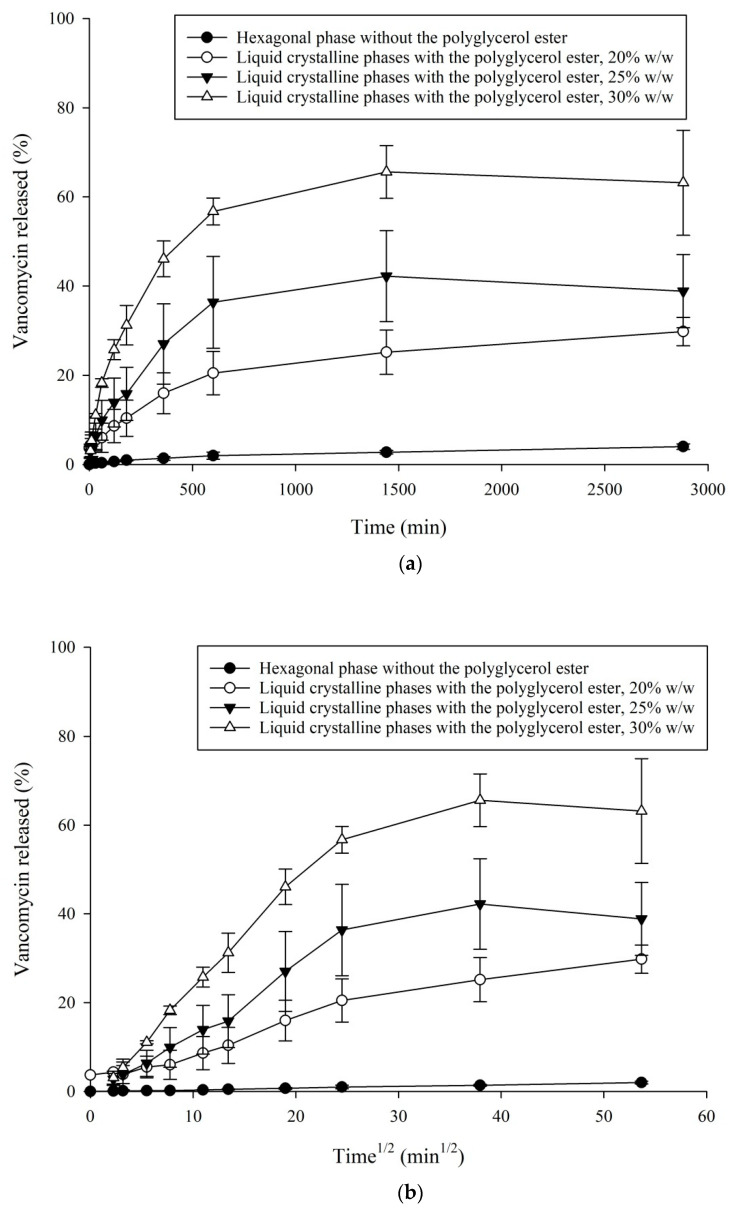
In vitro VHCl release test I at 32 °C from the LCPs tuned with PE, values are expressed as: (**a**) % VHCl released vs. time (min) and (**b**) % VHCl released vs. time^½^ (min^1/2^).

**Figure 4:**
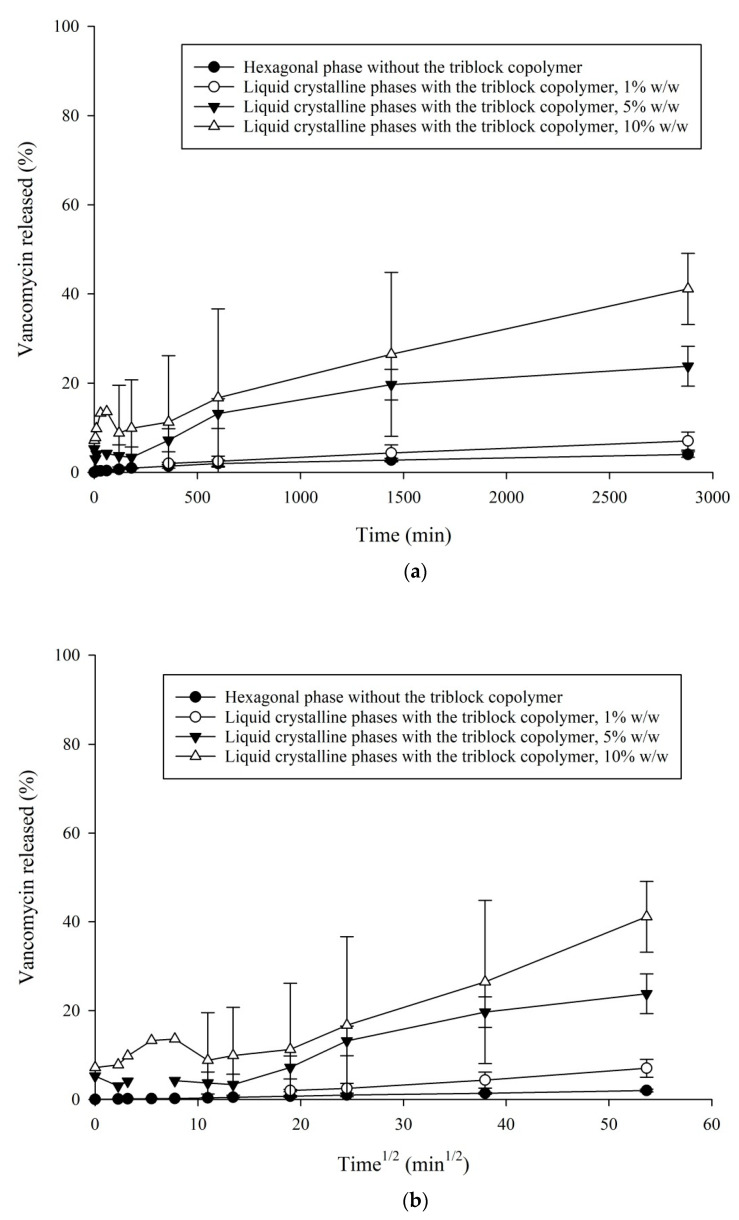
In-vitro VHCl release test I at 32 °C from the LCPs tuned with TC, values are expressed as: (**a**) % VHCl released vs. time (min) and (**b**) % VHCl released vs. time^½^ (min^1/2^).

Figure 3 and Figure 4 illustrating the LCPs with the tuning agents, PE and TC, demonstrate a significant increase in the VHCl release (with 30% *w*/*w* PE 65.61 ± 4.8% of VHCl, and with 10% *w*/*w* TC 26.46 ± 15.0% of VHCl) in comparison to the LCP without any tuning agent (2.73 ± 0.3% VHCl released after 1440 min (24 h) [37].

Increasing the PE conc. From 20% to 30% *w*/*w*, there appeared to be a significant monotonous increase in the amount of VHCl released. The maximum VHCl concentration was reached at 1440 min by the LCP tuned with PE 30% *w*/*w*, 65.61 ± 4.8% of VHCl; then the release seemed to decrease slowly (at 2880 min, 63.18 ± 9.6% of VHCl). The LCP with 10% *w*/*w* TC revealed a maximum VHCl concentration of 41.12 ± 6.5% after 2880 min.

At the early time point, the VHCl’s release data demonstrated a linear relationship (Figure 3b and Figure 4b), indicating that diffusion was the dominant release mode [40,42]. The correlation coefficient (R^2^) for the LCPs with PE ranged from 0.9327–0.9538, and for the LCPs with TC 0.8973–0.9832.

### 3.4. In Vitro VHCl Release Test II

**Figure 5:**
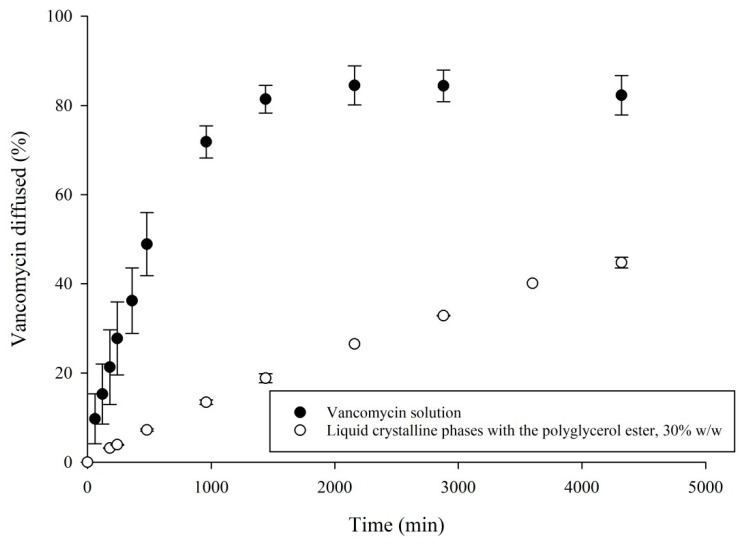
VHCl’s LCP tuned with PE (30% *w*/*w*) and VHCl solution analyzed by the in vitro VHCl release test II. The release rate of VHCl from the injection chamber following the introduction of VHCl’s LCP formulation in the presence of 5 mg/mL hyaluronic acid at 32 °C. The data represents the average (*n* = 3) ± S.D.

The permeation patterns of the VHCl solution and LCP tuned with PE, 30% *w*/*w*, have been revealed in Figure 5.

The VHCl solution revealed 81.40 ± 2.5% VHCl diffusional release into the carbonate buffer during the first 1440 min. However, the VHCl´s LCP (loaded with 9.5% *w*/*w* VHCl solution and PE 30% *w*/*w*) showed a release of 18.8 ± 0.8% during 1440 min. The VHCl´s LCP modulated with PE showed a total diffusion of 44.75 ± 0.9% into a carbonate buffer after 4320 min. The presence of an ECM decreased the fraction diffused compared to the diffusional rate in the simulated tear fluid (in vitro VHCl release test I) (Figure 3).

## 4. Discussion

The structure modulation of MO LCP has been investigated in an attempt to achieve the desired therapeutic VHCl concentration into the eye topically during 60 min and intravitreally during 2880 min using an LCP structure originated from the hexagonal phase, as described previously in our work [37] and the tuning agent, PE or TC.

A few techniques were applied to the characterization of LCPs—PLM, SAXS, and in vitro release tests I and II. PLM was used as a quick screening method for the LCP identity [9,38]. Our PLM results of tuned LCPs revealed the existence of one LCP structure—hexagonal phase. The hexagonal phase identity by PLM was indicated by a birefringence [9,38] (Figure 2).

The structure of the tuned LCPs was subsequently investigated by SAXS. SAXS measurements were conducted to elucidate the effect of PE and TC on the structure parameter of the LCP made of MO/PO/VHCl and a tuning agent. The SAXS results indicated an increase in the structure parameter of the hexagonal phase in samples with PE, changing from 7.25 nm in the samples with 10% *w*/*w* of PE to 8.7 nm in the samples with 30% *w*/*w* of PE (Table 1). The same trend of increasing the radius of water channels of the hexagonal phase by increasing the PE concentration has been demonstrated (Table 1). The hexagonal phase in the samples with PE appeared together with a lamellar LCP whose structure parameters were constant at the PE concentrations investigated, 20–30% *w*/*w*. The amphiphilic PE intercalated within the interfacial lipid layer. Its larger head group compared to MO flattened the interfacial film, thus increasing the structural parameter of the hexagonal phase. Similar results were noted in the literature, for example, by introducing the highly hydrophilic molecule such as desmopressin [43] or tryptophan [21] into the hexagonal phase.

The hexagonal-lamellar phase transition by a PE could be further explained by the well-rationalized concept of the critical packing parameters (CPP) [1], expressed as *v*/*Al*, which is the ratio between the volume of the hydrophobic lipid tail, *v*, and the product of the cross-sectional lipid head area, *A*, and the lipid chain length, *l*. It is assumed that molecules with a very large hydrophilic head group such as the PE increase the average area of the head group at the MO-water interface, thereby decreasing the CPP value. Decreasing the CPP value can induce a change in the space group from the hexagonal phase to a less negative curvature such as the lamellar phase.

In the mixture with TC, the hexagonal phase existed together with the phase whose determination of the space group was not straightforward. The identification of additional phase was difficult due to the unfortunate overlap of peaks and its lower concentration in the mixture.

Opposite to the above-mentioned effect of PE on the structure of a hexagonal phase, the structure parameter of the LCP modulated with TC has decreased being changed from 6.7 nm in samples with 1% *w*/*w* TC to 6.1 nm at the samples with 10% *w*/*w* TC (Table 1). The same trend of decreasing the radius of water channels of the hexagonal phase by increasing the TC concentration has been demonstrated (Table 1).

It is assumed that the hydrophobic part of the TC probably interacted with the outer hydrophobic surface of the reverse cylindrical micelles. The trend of the structural parameter decrease has been described in the literature, for example, by increasing the TC concentration [44]. Interestingly, the hexagonal structure parameter did not stay constant upon TC addition as was found for the dispersed nanostructured particles [13].

In the literature, a transition of MO LCP into the cubic phase has been observed in the presence of TC and is described by the interaction and possible anchoring of the TC molecules into the internal nanostructure of MO [45,46].

The release pattern from the LLC modulated with PE has been shown in Figure 3. Increasing the concentration of PE led to a gradual increase in the VHCl% released. In 1440 min, the amount of VHCl released by the liquid crystal sample with 20% *w*/*w* of PE was 25.17 ± 4.1%, by the liquid crystal sample with 25% *w*/*w* of PE was 42.22 ± 8.3% and by the liquid crystal sample with 30% *w*/*w* of PE was 65.61 ± 4.8%. The gradual increase of VHCl released with the increased PE concentration could be correlated, as mentioned before by the SAXS results, with the structure of swollen water cylinders by an LCP (hexagonal) and decreased CPP leading to the more favorable negative curvature, such as the lamellar phase.

A similar trend of increasing the released VHCl was observed by the LCP with TC. Figure 4 demonstrates the release properties of the VHCl LCP modulated with TC into the simulated tear fluid. The used TC concentrations were lower (1–10% *w*/*w* TC) than the PE concentrations (20–30% *w*/*w* PE). As the TC concentration in the samples was increased, there was a slow increase of VHCl% released. The maximal VHCl release from the LCPs with 5% *w*/*w* and 10% *w*/*w* TC was achieved after 2880 min and was 23.81 ± 3.6% (sample with 5% *w*/*w* TC) and 41.12 ± 6.5% (sample with 10% *w*/*w* TC).

As our SAXS results revealed, decreasing the values of the structural parameters by increasing the TC concentration, it was assumed that TC as a large hydrophilic molecule increased the average area of the head group, thereby decreasing the CPP value. This might lead to the energetically favorable structure of a less negative curvature, such as a cubic phase. However, our SAXS results did not confirm the appearance of a cubic phase.

The VHCl release results in the simulated tear fluids during the first 2 h were in the range up to 26% of the total VHCl diffused from the LCP’s samples with PE and up to 16% of the total VHCl diffused from the LCP’s samples with TC. This quick initial release could be described by some VHCl presented on the gel/water interface.

Additionally, our in-vitro release results (in vitro VHCl release test I) (Figure 3 and Figure 4) could not reach the VHCl concentration of 100% at the last time points investigated. The maximum amount of VHCl released was 65.61 ± 4.8% of VHCl detected in the in vitro VHCl release test I from the LCP modulated with 30% *w*/*w* PE after 1440 min, and 41.12 ± 6.5% VHCl from the LCP modulated with 10% *w*/*w* TC after 2880 min. In the case of a PE, an incomplete release could be explained by possible VHCl and PE´s arrangements into the interfacial film (bilayer). This is supported by our SAXS data demonstrating the coexistence of a hexagonal phase together with a lamellar phase. It is assumed that PE is located mostly in the interfacial film (lipid bilayer) and contributes to the interfacial film flattening. TC, as a large amphiphilic molecule, could demonstrate anchoring of its hydrophobic part into the internal nanostructure of MO [45,46]. Furthermore, it is probable that VHCl, PE and TC, and MO as amphiphilic molecules might show some interaction.

An incomplete drug release from MO LCPs has been reported previously [3,4,47,48,49,50]. This was explained by drug binding [50], bilayer participation [3], or by oxidation/hydrolysis of the lipid molecule [47].

The LCPs without a tuning agent have been prepared at maximum water capacity [37]. By adding PE or TC, it is expected to see increased water adsorption, as the water channels are larger. It has been demonstrated that by adding PE, an increased water capacity has been reported in the micellar phase [51]. As the same water concentration [37] was used in the preparation of tuned LCP, without tuning agent, it is probable for some structure swelling at the beginning of the release trial. This might explain the little decay in the release of VHCl from LCP with TC.

Additionally, our samples have demonstrated some decrease in the VHCl released at the last time points. After 1440 min by the sample with 25% *w*/*w* and 30% *w*/*w* PE the amount of VHCl released into simulated tear fluids was slowly decreased. As there were no impurities detected in the chromatograms of these samples, the decreased VHCl´s concentration could be ascribed to adsorption. The adsorption could occur to polypropylene tubes in the case of the in vitro VHCl release test I.

Our in vitro VHCl release results (in vitro VHCl release test I) were compared with the literature data. By standard local fortified VHCl ophthalmic drops of 25 mg/mL, the amount of VHCl in the eye in 60 min was 1.25 mg (theoretical, without any clearance/metabolic processes) [52]. Taking into account the 20%/min tear turnover (most of the drug lost by this process), one can get the concentration of 0.048 µg/mL in 60 min for the standard local fortified VHCl eye drops 25 mg/mL. Using the same calculation of 20%/min tear turnover by our in vitro VHCl release results, we received the concentration of 0.23 ng/mL of VHCl in 60 min by the LCP modulated with 30% *w*/*w* PE. There is a need to further optimize the LCP (insert) system developed to be able to reach the desired topical VHCl concentration in the eye in 60 min.

In vitro VHCl release test II (Figure 5) revealed a slower VHCl diffusion into the simulated intraocular tissue than into simulated tear fluid. The percentage of VHCl diffused into an ECM was 18.8 ± 0.8% after 1440 min, and 32.85 ± 0.1 after 2880 min, compared to 65.61 ± 4.8% VHCl released after 1440 min, and 63.18 ± 9.6% released after 2880 min from the same LCP with PE 30% *w*/*w*, into the simulated tear fluids. A similar trend of a decreasing amount of VHCl released into the ECM compared to the simulated tear fluid was already observed in our previous work [37]. This could be explained by a very complex composition of an extracellular matrix (hyaluronic acid gel) [53], a higher viscosity of the ECM, and an interaction with molecules in the ECM. It has been assumed that the transport of molecules through the ECM could be governed by a different mechanism such as steric exclusion (if the size of molecules is close to the pore size of the ECM), and electrostatic interactions (if the size of molecules is below the pore size of the ECM) [53,54,55]. We assume that the VHCl´s diffusion mechanism through the ECM might be by an interaction with the ECM.

Our in vitro VHCl release results (in vitro VHCl release test II) were compared with the literature data. Ferencz et al. presented the VHCl intravitreal concentrations in the range 25–182 µg/mL during 2880 min using 1 mg intravitreal VHCl injection (solution) [56]. Our in vitro VHCl release test II of the LCP sample with 30% *w*/*w* PE demonstrated 32.9 μg/mL VHCl diffused in 2880 min (Figure 5). Therefore, our intravitreal system developed might be effective in vivo in 2880 min.

The VHCl LCPs tuned with PE and TC were revealed to have the following benefits against the same LCP without the tuning agent (pure hexagonal phase):

- the structure of the MO hexagonal phase can be tuned with PE and TC;

- controlling the structure of the hexagonal phase with PE and TC, different release properties of VHCl could be achieved;

- the LCP tuned with 30% *w*/*w* PE might be effective in vivo intravitreally as our in vitro results (in vitro VHCl release test II) are in the desired intravitreal VHCl conc. range during 2880 min in comparison to the literature data.

Further investigations are needed on the stability of the drug delivery systems at its storage condition and in the ocular milieu. Although the kinetic of the MO degradation processes by lipase in the eye is not completely known, these insights might be further investigated by an in vitro release test using the release medium with lipase.

## 5. Conclusions

The structure and release properties of the MO/PO/VHCl LCPs modulated with PE or TC were studied with the aim to achieve the VHCl therapeutic concentrations in the eye, topically during 60 min and intravitreally during 2880 min.

It was shown by SAXS that the inclusion of PE into the MO LCP provided a partial transition of a hexagonal phase into a lamellar phase. The inclusion of TC into the MO LCP demonstrated a partial transition of a hexagonal phase into a phase whose identification was not reliable. Tuned LCPs, with PE or TC, were demonstrated to achieve a faster release pattern than by a hexagonal phase without a tuning agent.

The in vitro release test II suggests that the insert prepared from tuned VHCl´s LCPs with 30% *w*/*w* PE has the potential to successfully deliver an effective dose of VHCl in vivo into the eye intravitreally during 2880 min. However, the insert prepared from VHCl´s LCP with 30% *w*/*w* PE should be further optimized to reach the effective topical VHCl conc. in the eye for 60 min.

## Figures and Tables

**Table 1:** Structure parameters obtained by SAXS in VHCl LCPs formulations at different concentrations of PE and TC at 25 °C and the radius of water channels in the hexagonal phase calculated by the Equation (1).

Hexagonal Phase without Tuning Agent	% Glycerol Monooleate	Structure Parameter * (nm)			Radius of Water Channels in The Hexagonal Phase (Calculated) (nm)
**Hexagonal Phase without tuning Agent** [37]		H_II_	L_α_	*Ia3d*	H_II_
	63	6538 ± 0.02(37)	/	/	3.70
LCP tuned with		structure parameter * (nm)			radius of water channels in the hexagonal phase (calculated) (nm)
PE% (*w*/*w*)		H_II_	L_α_	*Ia3d*	H_II_
10	53	7.253 ± 0.06	(4.965 ± 0.05)	12.1387 ± 0.05	4.63
20	43	7.625 ± 0.07	4.887 ± 0.01	ND	5.36
25	38	7.996 ± 0.07	4.913 ± 0.01	ND	5.86
30	33	8.700 ± 0.20	5.009 ± 0.04	ND	6.63
TC% (*w*/*w*)		H_II_	L_α_	*/*	H_II_
1	63	6.732 ± 0.02	ND **	/	3.81
5	63	6.379 ± 0.06	ND **	/	3.61
10	63	6.061 ± 0.04	ND **	/	3.43

* structure parameter is expressed as the mean values ± standard deviation. ** ND indicates that the particular phase under consideration was not detected based on a SAXS analysis.
